# Effects of black garlic on the pacemaker potentials of interstitial cells of Cajal in murine small intestine *in vitro* and on gastrointestinal motility *in vivo*

**DOI:** 10.1080/19768354.2022.2049640

**Published:** 2022-03-10

**Authors:** Suk Bae Moon, Na Ri Choi, Jeong Nam Kim, Min Ji Kwon, Bo-Sung Kim, Ki-Tae Ha, Eun Yeong Lim, Yun Tai Kim, Byung Joo Kim

**Affiliations:** a Department of Surgery, Kangwon National University School of Medicine, Chuncheon, Republic of Korea; b Division of Longevity and Biofunctional Medicine, Pusan National University School of Korean Medicine, Yangsan, Republic of Korea; c Department of Korean Medical Science, School of Korean Medicine and Healthy Aging Korean Medical Research Center, Pusan National University, Yangsan, Republic of Korea; d Division of Functional Food Research, Korea Food Research Institute, Wanju-gun, Republic of Korea; e Department of Food Biotechnology, Korea University of Science & Technology, Daejeon, Republic of Korea

**Keywords:** Black garlic, pacemaker potential, antioxidant, gastrointestinal motility, interstitial cells of Cajal

## Abstract

Black garlic (BG) is a newly explored food stuff obtained via fermentation of raw, healthy garlic, especially in Asian countries. Interstitial cells of Cajal (ICC) are the pacemaker cells of gastrointestinal (GI) motility. The purpose of this study was to investigate the effects of BG extract on the pacemaker potentials of the ICC in the small intestines of mice and the possibility of controlling GI motility. The antioxidant activity of BG extract was also investigated. The whole-cell electrophysiological method was used to measure pacemaker potentials of the ICC *in vitro*, whereas GI motility was measured using the intestinal transit rate (ITR) *in vivo*. BG extract depolarized the pacemaker potentials of the ICC. Y25130 and RS39604 5-HT receptor antagonists could not inhibit the effect of BG extract on the pacemaker potentials of the ICC, whereas the 5-HT receptor antagonist SB269970 could. Pre-treatment with external Na^+^ (5 mM) or Ca^2+^-free solution inhibited the BG extract-induced depolarization of the ICC. With SB203580, PD98059, or c-jun NH_2_-terminal kinase II inhibitor pre-treatment, BG extract did not induce pacemaker potential depolarization. Moreover, the ITR values were increased by BG extract. Elevation of the ITR due to BG extract was related with increased protein expression of the 5-HT_7_ receptors. In addition, BG extract showed antioxidant activity. Collectively, these results highlight the ability of BG extract to regulate GI motility and the possibility of using it to develop GI motility modulators in the future. Moreover, BG showed immense potential as an antioxidant.

## Introduction

Garlic (*Allium sativum* L.) has been used as a spice and traditional medicine for eons. Several studies have shown that garlic has beneficial effects on human health, such as anti-inflammatory, anti-cancer, lipid lowering, maintenance of blood pressure, and blood glucose regulation (Kimura et al. 2017). However, unprocessed, raw garlic has a characteristic odor and spicy taste, which can limit its use because of gastrointestinal (GI) problems when consumed (Kodera et al. 2002). Black garlic (BG) is obtained after garlic has been fermented for a certain duration under high humidity and temperature conditions (Kimura et al. 2017). BG does not produce a strong off-flavor caused by the reduction of allicin, which converts it to an antioxidant during processing (Yuan et al. 2016). BG extract has demonstrated several bioactivities, including anti-oxidative, anti-allergic, anti-diabetes, anti-inflammation, anti-carcinogenic, and GI emptying effects (Jeong et al. 2016; Chen et al. 2018).

Interstitial cells of Cajal (ICC) are essential pacemaker cells that regulate the GI motility (Huizinga et al. 1995; Ward et al. 2000; Kim et al. 2005; Hwang et al. 2020. Kim et al, 2021). Thus, research on ICC plays a very important role in understanding GI motility regulation. However, little is reported on the effects of BG extract on ICC and GI motility. Thus, we investigated the effects of BG extract on the pacemaker potentials of ICC and GI motility. In addition, we assessed the antioxidant activity of BG extract.

## Materials and methods

### Preparation of the BG extract

BG was purchased from Taewoo Food Co. (Daejeon, Korea). A total of 2 kg of BG was extracted in 70% ethyl alcohol (20 L) for 6 h at 80°C and filtered through a Whatman No. 4 filter paper. After concentrating the solvents using rotary evaporation at 50°C, the yield was approximately 19.8% on a dry weight (w/w) basis.

### Preparation of ICC

Small intestines of mice were excised and after removing the mucous membrane, cut it into pieces. The cells were dispersed in a solution containing various enzymes, including collagenase, and were cultured in smooth muscle growth medium (Clonetics, San Diego, CA, U.S.A.) supplemented with a murine stem cell factor (Sigma-Aldrich, St. Louis, MO, U.S.A.) in a 95% O_2_ incubator.

### Electrophysiological experiments

A physiological salt solution (KCl 5, NaCl 135, CaCl_2_ 2, glucose 10, MgCl_2_ 1.2, and HEPES 10) was used to culture clusters of ICC. To examine the pacemaker activity, a solution comprising KCl 140, MgCl_2_ 5, K_2_ATP 2.7, NaGTP 0.1, creatine phosphate disodium 2.5, HEPES 5, and EGTA 0.1 was added to the cultured clusters of the ICC using a pipette. Whole-cell configuration was performed using an Axopatch 200B amplifier (Axon Instruments, Foster, CA, U.S.A.).

### Intestinal transit rate (ITR)

Evans blue (5%, w/v) was administered to healthy ICR mice after administration of BG extract into the stomach. After 30 min of Evans blue administration, the ITR of the mice was checked.

### GI motility dysfunction (GMD) model mice

We generated the GMD mouse models by using the acetic acid (AA, 0.6%, w/v, in saline)-induced peritoneal stimulation, as previously described (Lyu and Lee 2013). AA was injected intraperitoneally and the research process was carried out as described previously (Kim et al. 2013; Wu et al. 2013).

### Western blotting

After the proteins were separated, they were transferred on a polyvinylidene difluoride (PVDF) membrane. After 1 h of incubation with a blocking buffer, PVDF membranes were probed with anti-5-HT_3_ receptor (Abcam, Cambridge, UK), anti-5-HT_4_ receptor (Abcam, Cambridge, UK), anti-5-HT_7_ receptor (Mybiosource, San Diego, CA, U.S.A.), and anti-β-actin (Santa Cruz Biotechnology, Dallas, TX, U.S.A.) antibodies. Other experiments are the same as in previous studies (Han et al. 2021).

### Animals

Forty-nine mice (24 male and 25 female; 3–8-d-old) from ICR were used for the ICC experiments. In addition, 39 mice (male; 5–6-week-old) were used for the ITR experiments on heathy mice and mice with GI motility disease, whereas 10 mice (male; 5–6-week-old) were used for the protein expression experiments. The ICC and ITR experiments were completed within 12 h of culturing, respectively. We experimented according to The Institutional Animal Care and Use Committee at Pusan National University approved (approval no. PNU-2019-2462). Also, animals were handled according to the Guide for the Care and Use of Laboratory Animals.

### Reactive oxygen species (ROS) scavenging activity

A 0.2-mM 1,1-diphenyl-2-picrylhydrazyl (DPPH) solution was prepared by dissolving DPPH reagent in EtOH. The prepared solution and BG extract were mixed in a 1:1 ratio and incubated for 30 min in a dark room. Absorbance at 517 nm was measured and DPPH radical scavenging activity was calculated using the following formula:

$$DPPH\;radical\;scavenging\;activity\;\left(\text{\%}\right)=\left(1-\frac{A}{B}\right)\times 100,$$

where A is the absorbance value of the sample solution and B is the absorbance value of the control solution.

### Drugs

PD98059 and SB203580 were purchased from Tocris Bioscience (United Kingdom), whereas c-jun NH_2_-terminal kinase (JNK) II inhibitor was purchased from Calbiochem (San Diego, CA, U.S.A.). All other agents were purchased from Sigma-Aldrich.

### Statistical analyses

Data are expressed as the mean ± standard error of the mean. Significant differences were evaluated using one-way analysis of variance (ANOVA) or the Student’s *t*-test. *P* values < 0.05 were considered as significant.

## Results

### Effects of BG extract on the pacemaker potentials of ICC

**Figure 1.**
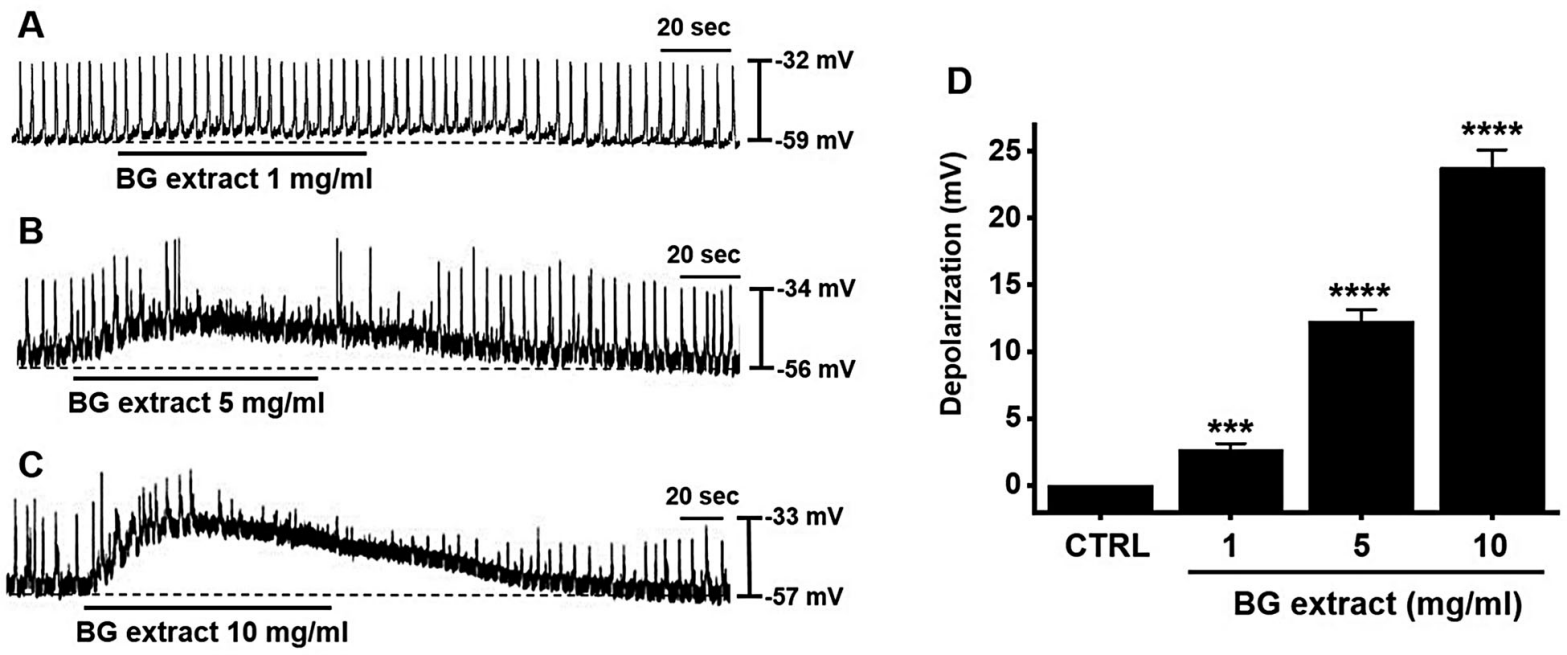
Effects of BG extract on the ICC pacemaker potentials from murine small intestines. (A–C) BG extract depolarized the ICC pacemaker potentials in a dose-dependently. (D) The changes of pacemaker potential depolarization caused by BG extract are summarized. Mean ± SEs. **P < 0.01. BG: Black garlic. CTRL: Control.

The whole-cell techniques showed that ICC spontaneously induced pacemaker potentials with an average resting membrane potential of –56.7 ± 1.4 mV (n = 164; Figure 1). BG extract (1–10 mg/mL) depo­larized the pacemaker potentials (Figure 1A–1C). The average depolarization were 2.7 ± 0.4 mV (n = 11; *P* < 0.001) at 1 mg/mL, 12.3 ± 0.9 mV (n = 10; *P* < 0.0001) at 5 mg/mL, and 23.8 ± 1.4 mV (n = 12; *P* < 0.0001) at 10 mg/mL (Figure 1D). These results indicated that BG extract depolarized the pacemaker potentials of ICC.

### Importance of the 5-HT_7_ receptors in BG extract-induced pacemaker potential depolarization of ICC

**Figure 2.**
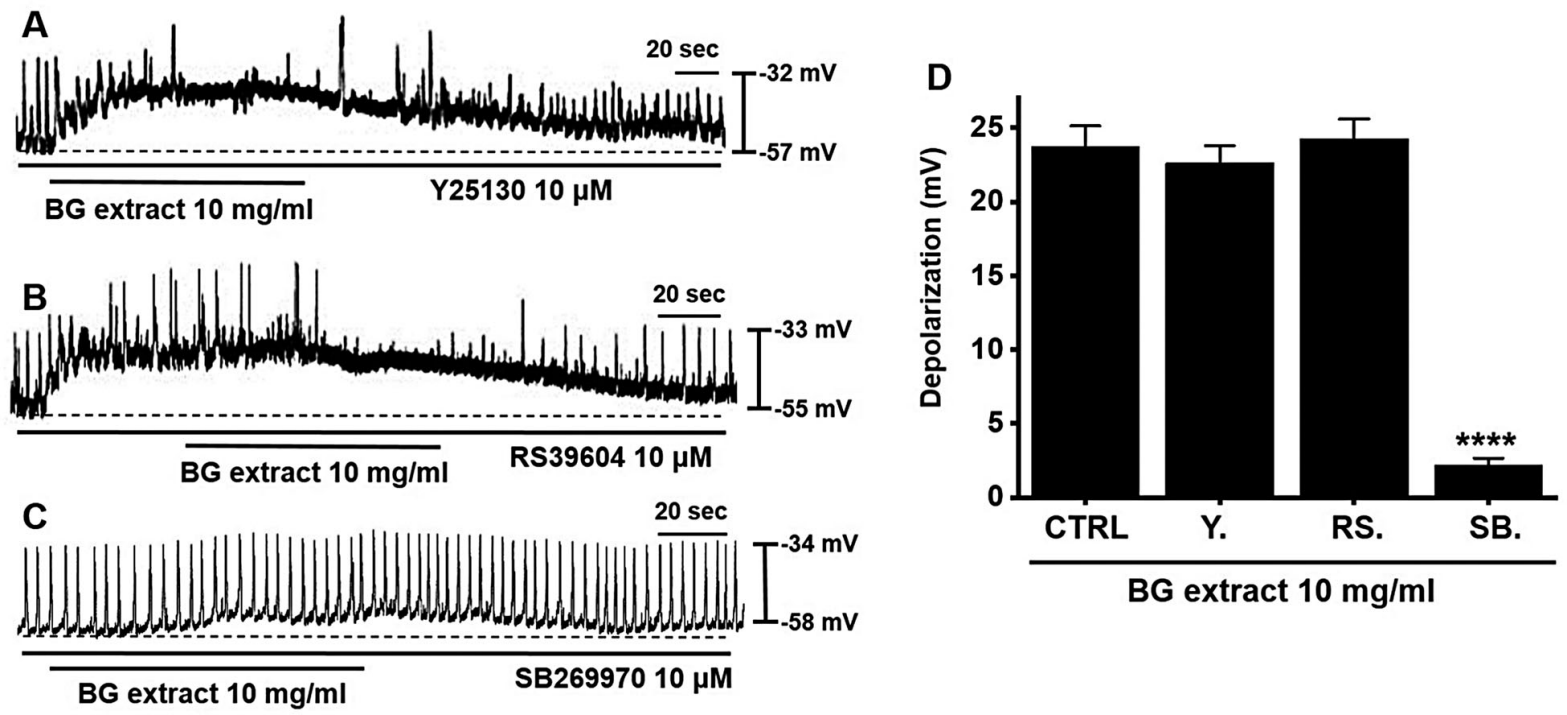
Effects of 5-HT receptor antagonists on BG extract-induced ICC pacemaker potential depolarization. (A and C) Pre-treatment with Y25130 or SB269970 and BG extract depolarized the ICC pacemaker potentials. (B) After pre-treatment with RS39604, BG extract did not depolarize. (D) Responses to BG extract are summarized. Mean ± SEs. **P < 0.01. BG: Black garlic. CTRL: Control. Y: Y25130. RS: RS39604. SB: SB269970.

Previous studies have shown that only three receptors (5-HT_3_, _4_, and _7_) were identified in the murine small intestinal ICC (Liu et al. 2011; Shahi et al. 2011). To check the involvement of 5-HT receptors, we exposed ICC to 5-HT receptor antagonists, namely, Y25130 (a 5-HT_3_ receptor antagonist), RS39604 (a 5-HT_4_ receptor antagonist), and SB269970 (a 5-HT_7_ receptor antagonist). Y25130 and RS39604 did not inhibit BG extract-induced responses (Figure 2A and 2B). However, SB269970 inhibited BG extract-induced responses (Figure 2C). The average depolarization were 22.6 ± 1.4 mV (n = 12) with Y25130, 24.3 ± 1.5 mV (n = 10) with RS39604, and 2.2 ± 0.5 mV (n = 12; *P* < 0.0001) with SB269970 treatment (Figure 2D). These results indicated that BG extract affected the pacemaker potentials in the ICC via the 5-HT_7_ receptors.

### Importance of extracellular Na^+^ and Ca^2+^ in BG extract-induced pacemaker potential depolarization of ICC

**Figure 3.**
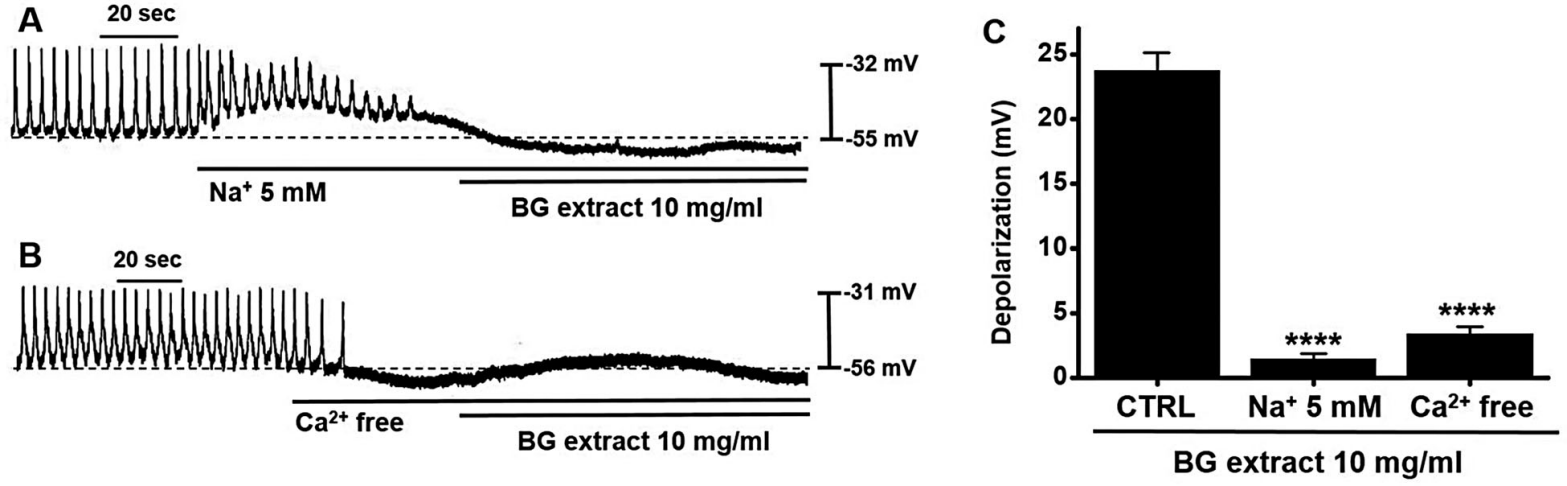
Effects of external Na^+^ (5 mM) or Ca^2+^-free solution on BG extract-induced ICC pacemaker potential depolarization. (A and B) In case of external Na^+^ or Ca^2+^-free solution, BG extract did not depolarize the pacemaker potential. (C) Responses to BG extract are summarized. Mean ± SEs. **P < 0.01. BG: Black garlic. CTRL: Control.

Both external Na^+^ and Ca^2+^ play a key role in regulating GI motility (Ward et al. 2000). To investigate the importance of external Na^+^ or Ca^2+^ in the BG extract-induced responses, we used external Na^+^ (5 mM) or Ca^2+^-free conditions. Pre-treatment with the external Na^+^ or Ca^2+^-free solution suppressed the pacemaker potentials and inhibited BG extract-induced responses (Figure 3A and 3B). The average de­grees of depolarization were 1.4 ± 0.5 mV (n = 9; *P* < 0.0001) with Na^+^ solution and 3.4 ± 0.6 mV (n = 13; *P* < 0.0001) with Ca^2+^-free solution (Figure 3C). These results indicated that the BG extract-induced response was controlled by external Na^+^ or Ca^2+^.

### Importance of mitogen-activated protein kinase (MAPK) in BG extract-induced pacemaker potential depolarization of ICC

**Figure 4.**
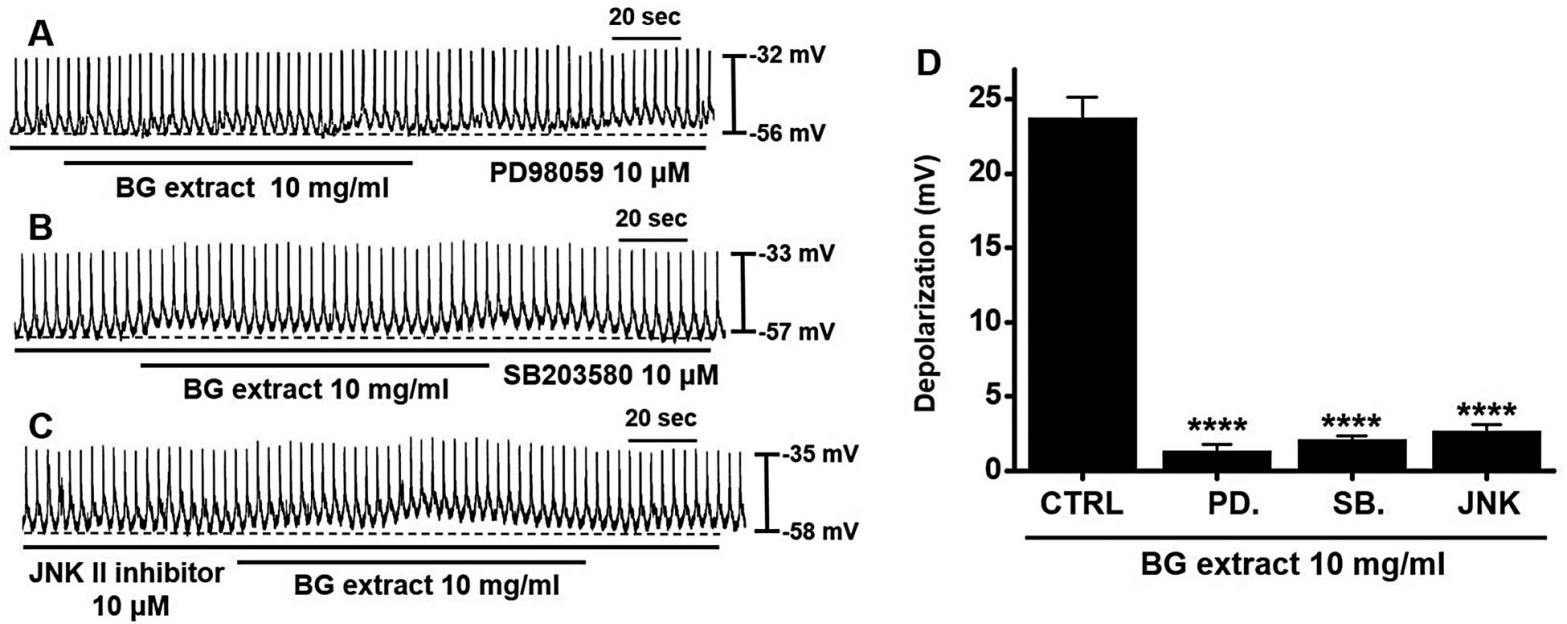
Effects of MAPK inhibitors on BG extract-induced ICC pacemaker potential depolarization. After pre-treatment with (A) PD98059, (B) SB203580, and (C) JNK II inhibitors, BG extract did not depolarize. (D) Responses to BG extract are summarized. Mean ± SEs. **P < 0.01. BG: Black garlic. CTRL: Control. PD: PD98059. SB: SB203580. JNK: JNK II inhibitor.

It has been reported that MAPK regulates the proliferation and differentiation of the GI tract (Jeong et al. 2012). Therefore, we assessed whether MAPK signaling affects the efficacy of BG extract on pacemaker potentials by treatment with PD98059 (a p42/44 inhibitor), SB203580 (a p38 inhibitor), and JNK II inhibitor. With PD98059 (n = 11), SB203580 (n = 10), or JNK II (n = 10) inhibitor treatment, BG extract did not induce the pacemaker potential depolarization (Figure 4). These results indicated that the BG extract-induced response was dependent on MAPK signaling.

### Effects of BG extract on the ITR

**Figure 5.**
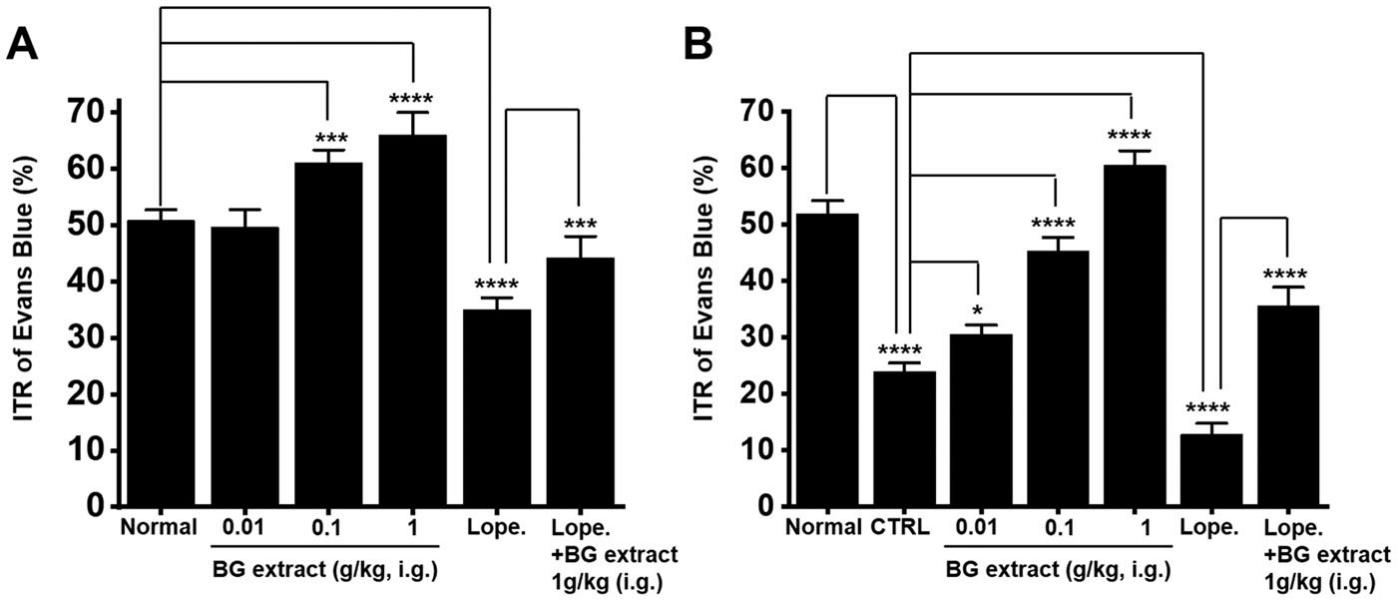
Effects of BG extract on the ITR in healthy and acetic acid-induced GMD mice. (A) BG extract increased the ITR. (B) The ITR was recovered by BG extract in acetic acid-induced GMD mice. Mean ± SEs. *P < 0.05. ***P < 0.001. ****P < 0.0001. BG: Black garlic. CTRL: Control. Lope: Loperamide.

In mice, the ITR was 50.8 ± 1.9% (n = 10; Figure 5A). BG extract increased the ITR to 49.6 ± 3.1% (n = 10) at 0.01 g/kg, 61.1 ± 2.3% (n = 10; *p* < 0.0001) at 0.1 g/kg, and 66.0 ± 3.9% (n = 10; *p* < 0.0001) at 1 g/kg (Figure 5A). Loperamide reduced the ITR, and BG extract recovered this loperamide-induced ITR reduction. For loperamide, the ITR was 35.1 ± 2.1% (n = 10; *p* < 0.0001), whereas for loperamide and BG extract, the ITR was 44.2 ± 3.8% (n = 10; *p* < 0.01), Figure 5A. These results indicated that BG extract increased the ITR.

### Effects of BG extract on the ITR in mice with GMD

We generated the GMD mouse model. AA decreased the ITR (23.9 ± 1.5% (n = 10) vs. 51.9% ± 2.3% (n = 10) in normal mice; *P* < 0.0001; Figure 5B). However, BG extract at 0.01, 0.1, and 1 g/kg restored this response to 30.5 ± 1.7% (n = 10; *P* < 0.05), 45.3 ± 2.4% (n = 13; *P* < 0.0001), and 60.5 ± 2.6% (n = 10; *P* < 0.0001), respectively (Figure 5B). Loperamide reduced the ITR in GMD mice to 12.8 ± 2.0% (n = 12; *P* < 0.0001) and BG extract recovered this value to 35.6 ± 3.2% (n = 11; *P* < 0.0001) (Figure 5B). These results indicated that BG extract recovered the ITR in GMD mice.

### Regulation of BG extract-induced small intestinal 5-HT receptor expression

**Figure 6.**
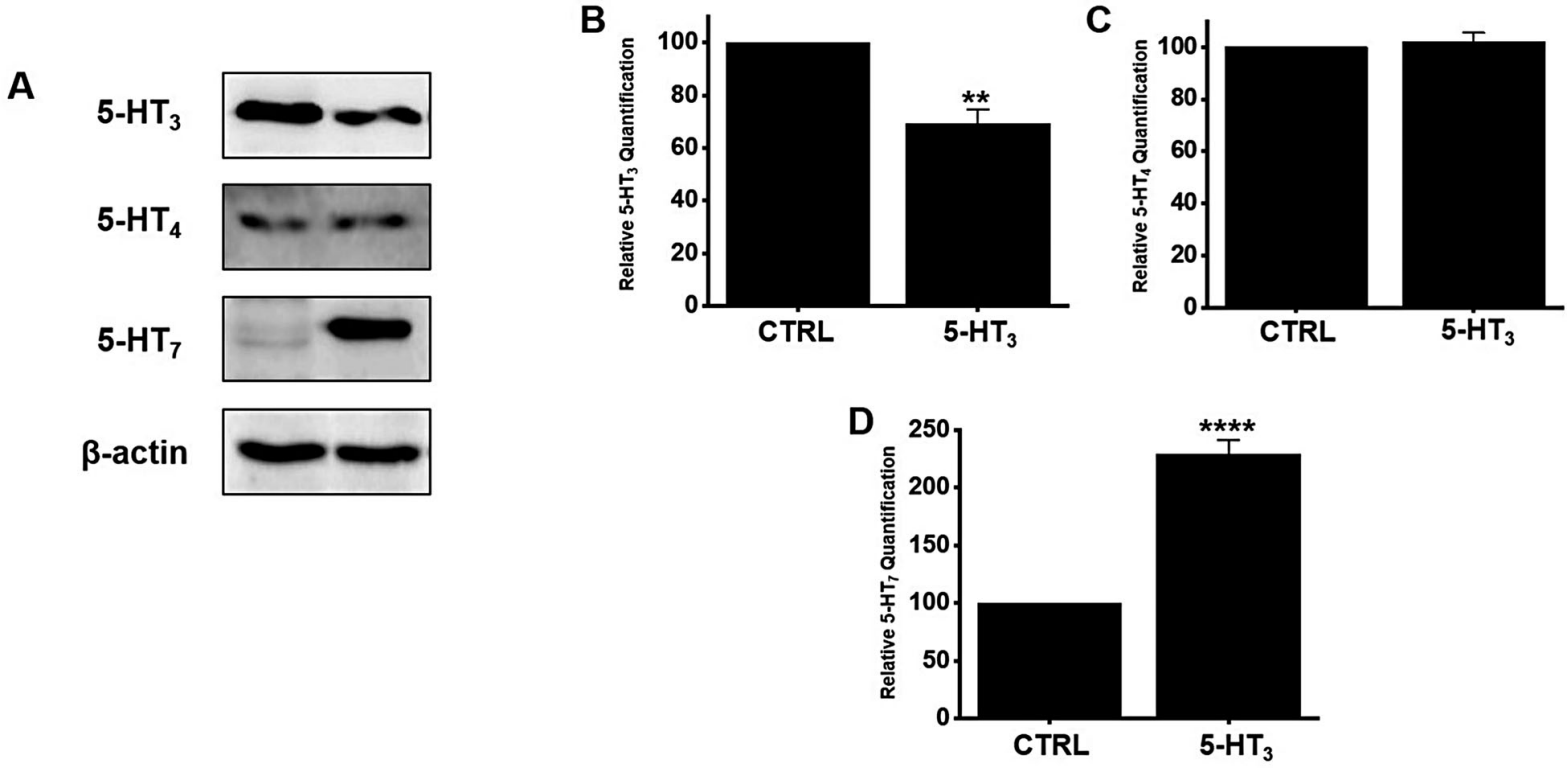
Effects of BG extract on the protein expression of 5-HT_3_, _4_, and _7_ receptors in mice. (A) 5-HT_7_ receptor expression increased considerably, but 5-HT_4_ receptors was unchanged. However, the expression of 5-HT_3_ receptors decreased. (B-D) Band density is showed relative to CTRL. Mean ± SEs. **P < 0.01. ****P < 0.0001. BG: Black garlic. CTRL: Control. β-Actin was the loading control.

5-HT is mainly present in the GI tract, and an increase or decrease in the expression of 5-HT directly affects GI motility (Camilleri 2009). Moreover, 5-HT_3_, _4,_ and _7_ receptors were found in ICC (Liu et al. 2011; Shahi et al. 2011). When BG extract was used, the expression of 5-HT_3_ receptors decreased significantly, the expression of 5-HT_4_ receptors did not change, and the expression of 5-HT_7_ receptors increased significantly (Figure 6A). The expression of 5-HT_3_ receptors decreased by 69.3 ± 5.4% (n = 5; *P* < 0.01) whereas that of 5-HT_7_ receptors increased by 228.9 ± 12.3% (n = 7; *P* < 0.0001) after BG extract treatment (Figure 6B and 6D). However, the expression of 5-HT_4_ receptors was unchanged (n = 6; Figure 6C). These results suggested that the ITR increase by BG extract was done by an increase in the expression of the 5-HT_7_ receptors.

### DPPH radical scavenging activity of BG extract

**Figure 7.**
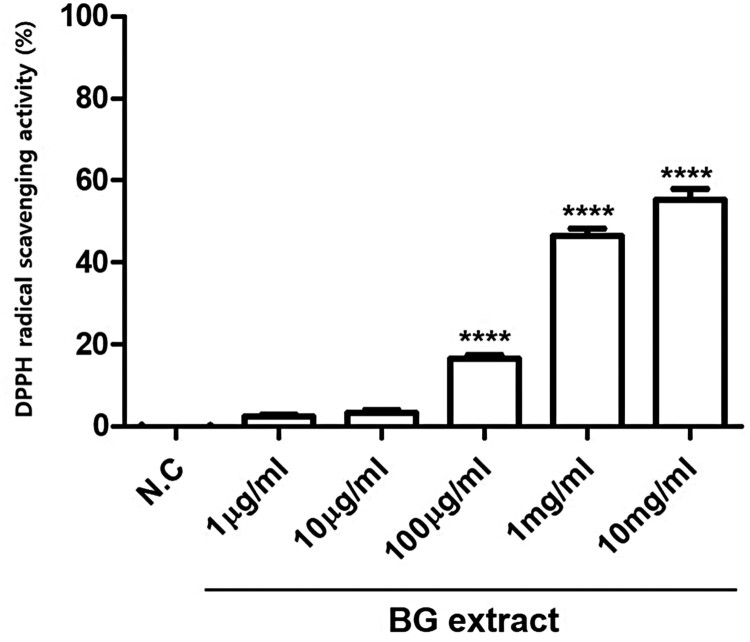
ROS scavenging activity was measured using the DPPH reagent. BG extract was treated with 0.001, 0.01, 0.1, 1, and 10 mg/mL DPPH reagent, and 100% EtOH was used as a negative control (N.C). The results are from three independent experiments. Mean ± SEs. ****P < 0.0001. BG: Black garlic. DPPH: 1,1-diphenyl-2-picrylhydrazyl.

To investigate the antioxidant activity of BG extract, ROS scavenging activity was measured using the DPPH assay. BG extract significantly exhibited ROS scavenging activity (16.6 ± 4.6% (n = 7; *P* < 0.001) at 0.1 mg/mL, 46.4 ± 9.5% (n = 7; *P* < 0.001) at 1 mg/mL, and 55. 4 ± 11.4% (n = 7; *P* < 0.001) at 10 mg/mL; Figure 7). These results suggested that BG extract has significant antioxidative effects.

## Discussion

Garlic has been used as a medicinal ingredient for a long time (Jeong et al. 2012). BG is a processed food, where fresh garlic is fermented at high humidity and high temperatures for 60–90 d (Kim et al. 2012; Yang et al. 2019). Although BG has various activities, including influencing GI motility (Jeong et al. 2016; Chen et al. 2018), its effect on the regulation of ICC function has not been reported yet. The ingredients of BG extract may change for various reasons, such as the type of solvent used for extraction. However, in this experiment, instead of experimenting with extracts of several batches, the experiment was conducted with the extract of one batch. Although no analysis of the components of the BG extract was conducted, the results of previous studies showed that, as compared to regular garlic, BG contained large amounts of diallyl trisulfide and allyl methyl trisulfide and a small amount of epicatechin (Martínez-Casas et al. 2017). Furthermore, it has been reported that lactic acid is a major organic acid component of BG extract (Lu et al. 2017). Another study also showed that BG contains S-allyl-L-cysteine, S-allylmercaptocysteine, pyruvate, and amino acids (Kim et al. 2015). In this study, we checked the efficacy of BG extract but not the efficacy of the individual ingredients. We plan to conduct a study to reveal the effective ingredients of BG in the future. We found that BG extract modulated the ICC pacemaker potentials. BG extract depolarized the ICC pacemaker potentials (Figure 1). External Na^+^ (5 mM) or Ca^2+^-free solution inhibited BG extract-induced pacemaker depolarization of ICC (Figure 3). BG extract increased the ITR. It also recovered the loperamide-induced decrease in ITR *in vivo* (Figure 5A). Moreover, BG extract recovered the ITR in AA-induced GMD in mice (Figure 5B). Therefore, it is thought that BG may control GI motility through the adjustment of the pacemaker potential of the ICC.

ICC generate spontaneously active pacemaker potentials (Huizinga et al. 1995). 5-HT is secreted from enterochromaffin cells present mostly in the gut. Liu et al. (2011) showed that the ICC pacemaker activity was controlled through 5-HT_3_ receptors, but Shahi et al. (2011) suggested that it was controlled through 5-HT_3_, _4_, and _7_ receptors. In addition, Wouters et al. (2007) stated that 5-HT_2B_ receptors regulate the growth of ICC. However, in this study, the 5-HT_7_ receptor antagonist SB269970 inhibited BG extract-induced responses, whereas the 5-HT_3_ and 5-HT_4_ receptor antagonists Y25130 and RS39604, respectively, did not. This shows that BG extract modulates pacemaker potentials due to the 5-HT_7_ receptors (Figure 2). In addition, BG extract-induced ITR increase was mediated by 5-HT_7_ receptors (Figure 6). Therefore, we hypothesize that 5-HT_7_ receptors play a vital role in the regulation of GI motility by BG extract. 5-HT_7_ receptors are present in lymphoid tissues, smooth muscle cells, ICC, and neurons within the gut (Tonini et al. 2005; Kim and Khan 2014). Various studies have demonstrated the relevance of 5-HT_7_ receptors in GI motility regulation (Tonini et al. 2005). In the future, we will study the mechanisms and roles of 5-HT_7_ receptors in GI motility and ICC in detail. In addition, MAPK signaling is also a major target for new treatments with GI motility disease (Ihara et al. 2011). In this study, MAPK inhibitors suppressed the effects of BG extract. It was observed that p38, p42/44, and JNK signaling is involved in the BG extract-mediated control of pacemaker potentials.

The human body has a variety of complex antioxidant defense mechanisms to counter the harmful effects of free radicals or other oxidants (Alam et al. 2013). Antioxidants are known to be very effective in preventing degenerative diseases and improving the quality of life (Alam et al. 2013). Compared to garlic, BG has approximately 10-fold stronger superoxide dismutase-like activity and antioxidant effects against hydrogen peroxide (Sato et al. 2006). In this study, we showed that the potent antioxidative effects of BG by using the DPPH radical scavenging assay (Figure 7).

Recently, natural herbal medicine has been attracting increasing attention as an alternative medicine with few side effects (Ekor 2014). Since many people have benefited from natural herbal medicine, we hope that research on the development of new treatments for GI diseases will be more active in the future.

**Figure 8.**
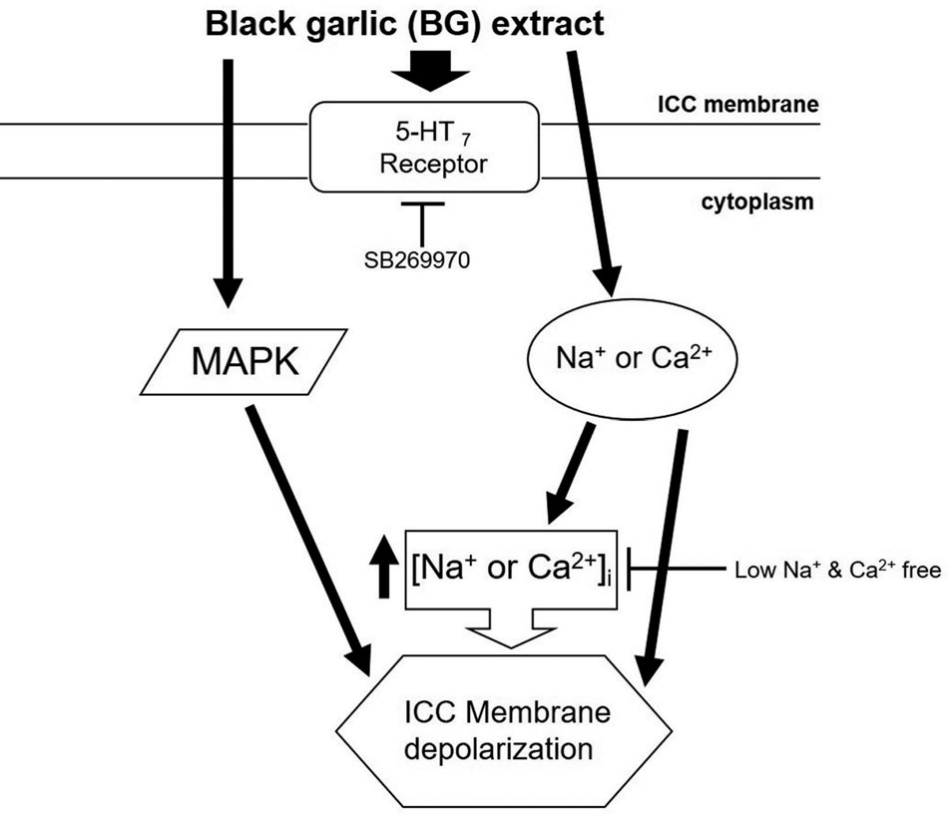
Schematic representation of the signaling pathway of BG extract-induced ICC depolarization. BG extract-induced depolarization may be mediated by 5-HT_7_ receptors, MAPK, and Na^+^ or Ca^2+^-dependent pathways. BG: Black garlic. ICC: Interstitial cells of Cajal.

Collectively, the results from the present study showed that BG extract depolarized the pacemaker potentials of the ICC via the 5-HT_7_ receptors, extracellular Na^+^ and Ca^2+^ concentration regulation, and MAPK pathways (Figure 8). Furthermore, BG extract increased the ITR in normal and GMD model mice. BG extract-induced ITR increase was mediated through the 5-HT_7_ receptors. In addition, BG extract showed significant antioxidative effects. Therefore, BG might be a prokinetic agent that can cure or prevent GMD, and herbal medicine may become a very important strategy for the treatment of GI tract disorders.
